# Overexpression of Specific CD44 Isoforms Is Associated with Aggressive Cell Features in Acquired Endocrine Resistance

**DOI:** 10.3389/fonc.2016.00145

**Published:** 2016-06-20

**Authors:** Rebecca Bellerby, Chris Smith, Sue Kyme, Julia Gee, Ursula Günthert, Andy Green, Emad Rakha, Peter Barrett-Lee, Stephen Hiscox

**Affiliations:** ^1^School of Pharmacy and Pharmaceutical Sciences, Cardiff University, Cardiff, UK; ^2^Institute of Pathology, University Hospital of Basel, Basel, Switzerland; ^3^Faculty of Medicine and Health Sciences, University of Nottingham, Nottingham, UK; ^4^Velindre Cancer Centre, Cardiff, UK

**Keywords:** CD44, breast cancer, endocrine resistance, invasion

## Abstract

While endocrine therapy is the mainstay of ER+ breast cancer, the clinical effectiveness of these agents is limited by the phenomenon of acquired resistance that is associated with disease relapse and poor prognosis. Our previous studies revealed that acquired resistance is accompanied by a gain in cellular invasion and migration and also that CD44 family proteins are overexpressed in the resistant phenotype. Given the association of CD44 with tumor progression, we hypothesized that its overexpression may act to promote the aggressive behavior of endocrine-resistant breast cancers. Here, we have investigated further the role of two specific CD44 isoforms, CD44v3 and CD44v6, in the endocrine-resistant phenotype. Our data revealed that overexpression of CD44v6, but not CD44v3, in endocrine-sensitive MCF-7 cells resulted in a gain in EGFR signaling, enhanced their endogenous invasive capacity, and attenuated their response to endocrine treatment. Suppression of CD44v6 in endocrine-resistant cell models was associated with a reduction in their invasive capacity. Our data suggest that upregulation of CD44v6 in acquired resistant breast cancer may contribute to a gain in the aggressive phenotype of these cells and loss of endocrine response through transactivation of the EGFR pathway. Future therapeutic targeting of CD44v6 may prove to be an effective strategy alongside EGFR-targeted agents in delaying/preventing acquired resistance in breast cancer.

## Introduction

Over two-thirds of breast cancers express the estrogen receptor and are, likely to respond to endocrine therapies exemplified by tamoxifen (1, 2). However, despite the undoubted improvements brought by such treatments, acquired resistance to these agents remains a significant problem with resistant tumors frequently recurring at distant sites (3). Increasingly, evidence suggests that a gain in aggressive, metastatic cell functions accompanies acquisition of resistance (4–7) although the mechanisms underlying this are unclear. Thus, a greater understanding of the molecular pathways associated with resistance is needed in order to highlight better therapeutic opportunities. To this end, we have previously reported that acquired tamoxifen resistance in breast cancer is accompanied by the overexpression of the CD44 transmembrane receptor protein that appears to contribute to their aggressive phenotype through modulation of growth factor receptor signaling.

A challenge to developing therapies against CD44 lies in the fact that CD44 represents a large family of variant isoforms expressed through alternative splicing of the CD44 gene and controversy exists as to which isoforms are tumor-protective and which may act to promote tumor progression (8, 9). However, the majority of studies appear to have focused on CD44v3 and CD44v6 and their role as promoters of tumor migration, invasion, and spread (10–13). CD44v6 is specifically implicated in the tumorigenesis and migration of tumor cells during metastasis (14, 15) while overexpression of CD44v6 is suggested to predict overall survival (OS) and disease-free survival (DFS) in breast and other cancers (16–18). A number of studies also support an association between CD44v3 and invasion (10) and metastasis (19, 20). Interestingly, in many of these cases, it is the co-expression of CD44 isoforms alongside other growth factor receptors that confers a poor prognostic signature.

While our previous study looked at global CD44 expression, identifying specific CD44 variants that may play a role in drug resistance represents an important goal with respect to identifying specific elements that may represent future therapeutic opportunities. In this study, we provide data supporting the hypothesis that the CD44v6 rather than CD44v3 acts to limit endocrine response and promote tumor progression through EGFR transactivation in breast cancer cells. Importantly, our translational studies further suggest that CD44v6 and EGFR co-expression may represent an important prognostic marker for ER+ breast cancers.

## Materials and Methods

### Cell Culture

Endocrine responsive MCF-7 cells were maintained in phenol-red free RPMI with l-glutamine (200 mM) containing 5% (v/v) fetal calf serum and antibiotics [penicillin (10 IU/ml), streptomycin (10 μg/ml), and fungizone (2.5 μg/ml)]. Tamoxifen-resistant (Tam-R) and fulvestrant-resistant (Fas-R) cells (4, 7) were cultured in phenol-red free RPMI containing 5% (v/v) charcoal-stripped steroid depleted fetal calf serum, antibiotics as above and supplemented with 4-hydroxy tamoxifen (100 mM) or fulvestrant (100 mM), respectively. Antibodies were used that recognized CD44 Std (Clone 156-3C11; Fisher Scientific), anti-CD44v3 (Clone VFF-327) and anti-CD44v6 (Clone 2F10) (both R&D Systems), and RHAMM (Abcam). All other antibodies were purchased from Cell Signaling Technologies. All other reagents were from Sigma Ltd. (Dorset, UK), unless otherwise stated.

### Microarray Analysis

Triplicate RNA samples from MCF-7, Tam-R, and Fas-R cells were microarrayed using Affymetrix Human Genome U133A gene chips with subsequent median normalization across all datasets and log transformation using Genesifter. Heatmap profiles and log2 intensity plots were generated to visualize gene expression changes across all cell models. The genes analyzed by microarray were as follows: CD44, HMMR (the gene corresponding to the RHAMM protein), stabilin 2 (STAB-2), lymphatic vessel endothelial receptor 1 (LYVE-1), toll-like receptor 4 (TL4), intracellular adhesion molecule-1 (ICAM-1), and versican (VCAN). Statistical analysis of probe expression between cells was performed using ANOVA with Tukey *post hoc* analysis.

### RT-PCR

mRNA was isolated from MCF-7, Tam-R, and Fas-R cells and reverse-transcribed to cDNA using primers corresponding to the standard form of CD44 (sF: 5′GACACATATTGGCTTCAATGCTTCAGC3′; sR: GATGCCAAGATGATCAGCCATTCTGGAAT3′), CD44 variant 3 (sF:5′ AGTCACAGACCTGCCCAATGCCTTT3′; sR: 5′GGTGTCTGTCTCTTTCATCTTCATTTTTCTTCATTT3′), variant 6 (sF: 5′ CAACGGAAGAAACAGCTACC3′; sR: 5′CCTGTTGTCGAATGGGAGTC3′), and β-actin (sF: 5′GGAGAATGATCTTGATCTT3′ sR 5′CCTTCCTTGGGCATGGAGTCCT3′). All PCRs were performed in a semi-quantitative manner using 27–30 cycles so that products were in a linear range of amplification. PCR products were separated and visualized on a 1% agarose gel using ethidium bromide and photographed (representative images are shown from a minimum of three separate experiments).

### Cell Lysis and Western Blotting

Log phase cultures were lysed in Triton X100 lysis buffer containing protease inhibitors. Clarified supernatants were boiled in sample buffer and equal amounts of proteins and resolved by 8% SDS-PAGE. Separated proteins were immobilized on nitrocellulose membranes and probed with antibodies against CD44 Std, CD44v6, CD44v3, RHAMM, and the activated forms of EGFR (Y1068), ErbB2 (Y1248), Met (Y1234/1235), FAK (Y397), MAPK (T202/Y204), AKT (S473), Src (Y416), and GAPDH. Bound primary antibodies were detected by HRP-conjugated secondary anti-mouse or anti-rabbit IgG (Fisher Scientific, UK) and subsequent chemiluminescence analysis. Representative blots are shown from a minimum of three separate experiments.

### Immunocytochemistry

Log-phase cultures of MCF-7, Tam-R, and Fas-R cells were fixed with 2.5% phenol formal saline and stained with primary antibodies. Antibody detection was performed with Dako mouse EnVision (peroxidase-labeled polymer) for 30 min and DAB chromogen, counterstaining with 1% methyl green. Photographs were taken of cells at ×40 magnification. Plasma membrane and cytoplasm percentage positivity were assessed to derive a total *H*-score value for each cell line.

### Immunohistochemical Analysis of CD44v6 Expression in Clinical Breast Cancer

Tumor tissue was available from patients with histologically proven breast cancer that presented for surgery at Nottingham City Hospital between 1984 and 1987. This series comprised part of an historical breast cancer collection with approval for use without further patient consent [approved ethics application C108030; Nottingham Research Ethics Committee (21)]. All patients received systemic tamoxifen therapy for locally advanced primary carcinoma (>5 cm) and the duration of anti-hormonal response and survival from the initiation of therapy monitored for each patient by follow-up after surgery. No patient had previously received any form of adjuvant endocrine or cytotoxic therapy; the median patient age was 54 years (age range 25–77 years). Survival and duration of anti-hormone response were measured from commencement of anti-hormone to death or progression on therapy, respectively. All patient-related information was anonymized and de-identified prior to analysis and the EGFR status of these tumors was previously reported (22). Paraffin-embedded tissue sections were subject to antigen retrieval by microwaving for 30 min in 0.1 M citrate buffer (pH 6). Following elimination of endogenous peroxide, sections were then blocked with 10% normal human serum in PBS for 10 min. Immunostaining was then performed using CD44v3 (1/80) and CD44v6 (1/40) antibodies for 2 h and antibody detection was performed with Dako mouse EnVision (peroxidase-labeled polymer) for 90 min and DAB chromogen for 10 min, counterstaining with 1% methyl green for 15 min. Negative controls were incubated with mouse isotype-specific control IgG (Dako). All sections were assessed simultaneously by two observers blinded to the clinical data using a dual-viewing light microscope (×40 magnification). Matched control slides were checked for non-specific binding before assessment of staining intensity. The data were then used to construct a CD44v6 *H*-score for each tumor specimen as described below. Statistical analysis [Log Rank (Mantel–Cox)] was used to investigate relationship between CD44v6 and EGFR and clinicopathological parameters using SPSS version 20. Kaplan–Meier survival curves were plotted with a log-rank test to analyze the differences between curves. The relative influence of CD44v6 and EGFR on survival was examined using multivariate analysis according to the non-parametric hazards model of Cox. Significance was set at *P* < 0.05.

### Evaluation of Immunohistochemical Staining

Staining intensity was evaluated using the *H*-score as follows: for each sample, six fields of view were investigated and allocated a value between 0 and 3 dependent on staining intensity (0 = negative staining, 3 = strong staining). A total *H*-score value was calculated using the formula: (% at 0) × 0 + (% at 1) × 1 + (% at 2) × 2 + (% at 3) × 3 to give an overall score ranging between 0 and 300. Differences between groups were statistically compared using the Student’s *t*-test. Representative images are shown from a minimum of three separate experiments.

### Analysis of Cell Migration

A total of 3 × 10^4^ cells were seeded into the top chambers of 24-well transwell plates (BD Biosciences, 8.0 μm pore size coated with 10 μg/ml fibronectin) with complete media added to the bottom chamber. After 24 h of culture, migratory cells (cells that had moved to the underside of the membrane) were fixed with 3.7% paraformaldehyde, stained with 0.5% crystal violet, and counted at ×40 magnification under a light microscope. Cell migration was quantified as the mean number of cells observed in each of five random fields of view per sample, each performed in duplicate over three independent experiments and then normalized to the untreated controls (i.e., expressed as a percentage of the cells migrating in the control samples). Differences between groups were statistically compared using the Student’s *t*-test.

### Boyden Chamber Invasion Assay

A total of 3 × 10^4^ cells were seeded onto Matrigel-coated (9.6 mg/ml) transwell chambers (8 μm pore size) with complete media in the bottom chamber. After 72 h of culture, the membranes from each transwell insert were removed to allow cells that had invaded through the Matrigel layer to the underside of the membrane to be fixed with 3.7% paraformaldehyde prior to mounting on microscope slides using DAPI-containing vector shield. Cell nuclei were counted at ×60 magnification using a fluorescent microscope. Cell invasion was quantified as above by counting the mean number of cells observed in each of five random fields of view per sample, each performed in duplicate and normalizing to controls (untreated samples). Differences between groups were statistically compared using the Student’s *t*-test.

### Coulter Counter Growth Assay

Cell proliferation was determined using the Coulter counter growth assay. Briefly, cells were seeded into 24-well plates at 1.5 × 10^6^ cells/plate and left to adhere for 24 h. Subsequently, fresh medium containing treatments as described were added and cells were cultured for a further 5 days. Media were then removed from the wells and replaced with 1 ml/well trypsin and returned to the incubator until cells were in suspension. Trypsin (4 ml/well) was added using a sterile syringe and this solution was added to 6 ml Isoton. The 11 ml Isoton cell suspension was counted twice on a Coulter counter (CoulterTM Multisizer II, UK) according to manufacturer’s instructions. The average count was multiplied by 20 to calculate cells/well. Differences between groups were statistically compared using the Student’s *t*-test.

### siRNA-Mediated Gene Suppression in Endocrine-Resistant Cells

siRNA knockdown of CD44 (all forms), CD44v6, and RHAMM was performed in Tam-R and Fas-R cells as follows. Briefly, log-phase Tam-R and Fas-R cells were inculbated with 100 nM siRNA/lipid mixture as per the manufacturer’s protocol (Dharmacon Ltd.) for 48 h. Suppression of gene and protein expression was confirmed using PCR and Western blotting. After gene suppression, cells were harvested and used in the assays described above. Control experiments used either non-targeting (NT) siRNA or transfection lipid only.

### Transient Transfection of Endocrine-Sensitive MCF-7 Cells with CD44v3 and CD44v6

Plasmid constructs containing cDNA corresponding to CD44v3 and CD44v6 isoforms (in a pPGK-T7/2 vector) were provided by Ursula Günthert, Basel University. These constructs were introduced into MCF-7 cells as follows. Briefly, cells at 50% confluency were incubated with transfection lipid alone (FuGENE 6, Promega, UK) or with transfection lipid plus the DNA plasmid construct (100 ng/well at 3:1 lipid:DNA ratio) in culture medium for 48 h after which cells were harvested and used in the assays described.

## Results

### CD44v3 and CD44v6 Are Overexpressed in Acquired Tamoxifen- and Fulvestrant-Resistant Breast Cancer Cells

**Figure 1:**
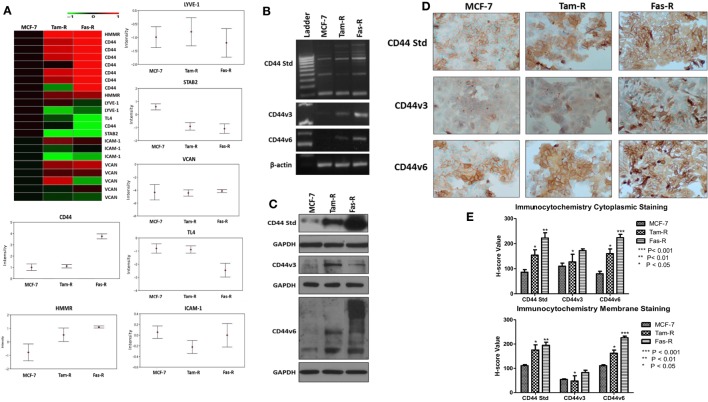
**CD44 isoforms are overexpressed in acquired tamoxifen- and fulvestrant-resistant breast cancer cells**. **(A)** Affymetrix U133A gene microarray analysis revealed deregulation of CD44 and its related family members LYVE-1, TL4, STAB-2, ICAM-1, and VCAN in acquired resistant cell models. Changes in CD44 gene expression were confirmed by RT-PCR **(B)**, Western blotting **(C)**, and immunocytochemistry/*H*-score analysis **(D,E)** that revealed consistent upregulation of CD44v3 and CD44v6 isoforms in resistant models.

An initial interrogation of our in-house microarray database was performed to identify members of the wider hyaladherin family, as well as CD44 itself, that may be deregulated in acquired resistance compared to their drug-sensitive counterparts (Figure 1A). These data pointed to an upregulation of both CD44 and receptor for HA-mediated motility (HMMR/RHAMM) in Tam-R and Fas-R models compared to MCF-7 cells, while expression of other hyaladherin members, LYVE-1, TL4, STAB-2, ICAM-1, and VCAN were suppressed. As the microarray probe sets could not differentiate between CD44 isoforms, RT-PCR was performed to explore whether specific CD44 isoforms (specifically CD44v3 and CD44v6) were altered in addition to global CD44 expression. RT-PCR confirmed the microarray data showing upregulation of global CD44 transcripts and further revealed elevated expression of CD44v3 and CD44v6 isoforms in the resistant models (Figure 1B). Elevated levels of CD44, v3, and v6 isoforms were subsequently confirmed at the protein level by Western blotting that revealed higher levels of CD44 Std (80 kDa), CD44v3 (85 kDa), and CD44v6 (85–200 kDa) in Tam-R and Fas-R cells versus their MCF-7 counterparts (Figure 1C). Immunocytochemical staining of CD44 Std and CD44v6 isoforms revealed predominant cell surface staining in contrast to CD44v3 proteins that revealed intense cytoplasmic staining (Figure 1D). Quantitation of staining by *H*-score revealed a CD44 distribution profile across the cell lines largely reflecting the Western data, with overexpression of CD44 and its variants in the resistant cells compared to their endocrine-sensitive counterparts. Cytoplasmic expression appeared similar although CD44v3 levels appeared only modestly elevated in Tam-R cells in contrast to the Western data (Figure 1E).

### Inhibition of CD44 Suppresses the Aggressive Phenotype of Endocrine-Resistant Breast Cancer Cells

**Figure 2:**
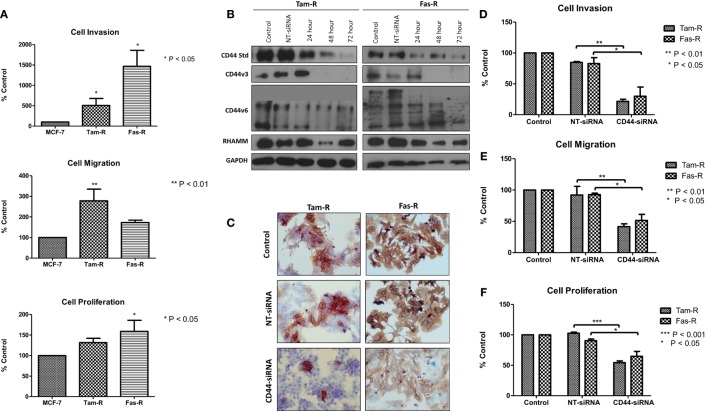
**siRNA-mediated CD44 suppression reduces the aggressive behavior of endocrine-resistant breast cancer cell models**. **(A)** Tam-R and Fas-R cells display enhanced migratory, invasive, and proliferative capacity compared to MCF-7 cells. **(B)** Western blotting demonstrated that treatment of Tam-R and Fas-R cells with CD44-siRNA resulted in suppression of CD44s, CD44v3, and CD44v6 isoforms and global CD44 loss was confirmed by immunocytochemistry **(C)**. Suppression of CD44 expression subsequently resulted in loss of invasive **(D)**, migratory **(E)**, and proliferative responses **(F)** in Tam-R and Fas-R cells.

Our previous research has shown that endocrine resistance is accompanied by aggressive cellular behavior (23) and confirmed in this study (Figure 2A). To begin to understand the contribution of CD44 variants to cellular phenotype in the context of acquired endocrine resistance, we first suppressed all forms of endogenous CD44 in Tam-R and Fas-R cells using siRNA (Figures 2B,C). This resulted in a reduction in the intrinsic migratory, proliferative, and invasive nature of both resistant cell lines (Figures 2D–F).

### CD44-siRNA Reduces EGFR and Met Pathway Activity in Tam-R and Fas-R Cells, Respectively

**Figure 3:**
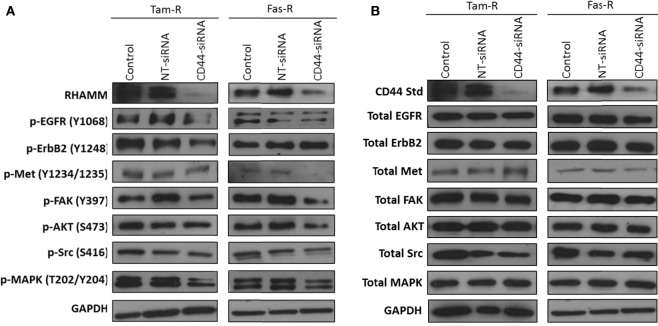
**Inhibition of CD44 expression suppresses endogenous growth factor pathway activity in Tam-R and Fas-R cells**. Western blotting analysis was used to determine the effects of CD44 suppression on signaling activity in Tam-R and Fas-R cells. **(A)** CD44 suppression reduced EGFR, ErbB2, and MAPK phosphorylation in Tam-R cells and reduced Met, AKT, and MAPK phosphorylation in Fas-R cells. **(B)** No change in total protein levels was observed.

Growth factor receptor pathway activity is suggested to play an important role in resistance with previous reports implicating the EGFR/HER2 (3, 24) and c-Met (7) pathways in acquired tamoxifen and fulvestrant resistance respectively where they may contribute to an enhanced migratory phenotype. Since CD44 can act as a co-receptor for receptor tyrosine kinases (25–27) we investigated whether signaling through these receptors was affected in the absence of CD44. Western blotting revealed that, following suppression of CD44 expression by siRNA, there was a reduction of endogenous EGFR and associated signaling pathway activity in Tam-R cells (Figure 3A). While no effects were seen on EGFR signaling in Fas-R cells, suppression of CD44 in these cells reduced the activity of the Met receptor (Figure 3) previously implicated in Fas-R cell migratory responses (7). Accompanying this decrease was a suppression of MAPK and AKT in both cell lines; no changes were observed in total levels of these proteins (Figure 3).

### RHAMM Does Not Contribute to the Aggressive Phenotype of Endocrine-Resistant Breast Cancer Cells

**Figure 4:**
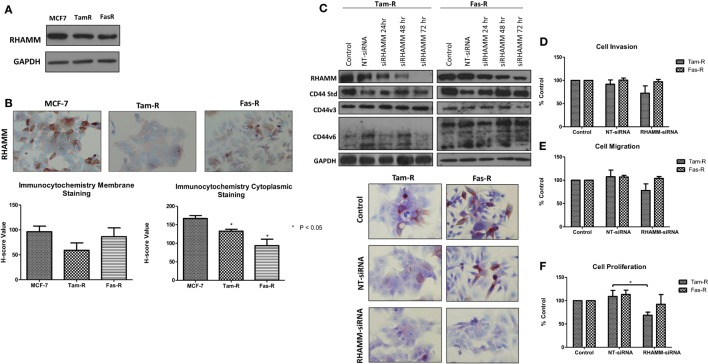
**Inhibition of RHAMM expression does not alter the aggressive behavior of endocrine-resistant breast cancer cells**. **(A)** Western blotting revealed a similar level of RHAMM protein expression across MCF-7, Tam-R, and Fas-R cells, which was confirmed by immunocytochemistry and *H*-score analysis. **(B)**. Suppression of RHAMM by siRNA **(C)**, confirmed with immunocytochemical staining and Western blotting, did not alter CD44 variant isoform expression. RHAMM suppression did not alter the invasive **(D)** or migratory **(E)** capacity of Tam-R and Fas-R cells although some suppression of Tam-R proliferation was observed in the absence of RHAMM **(F)**.

**Figure 5:**
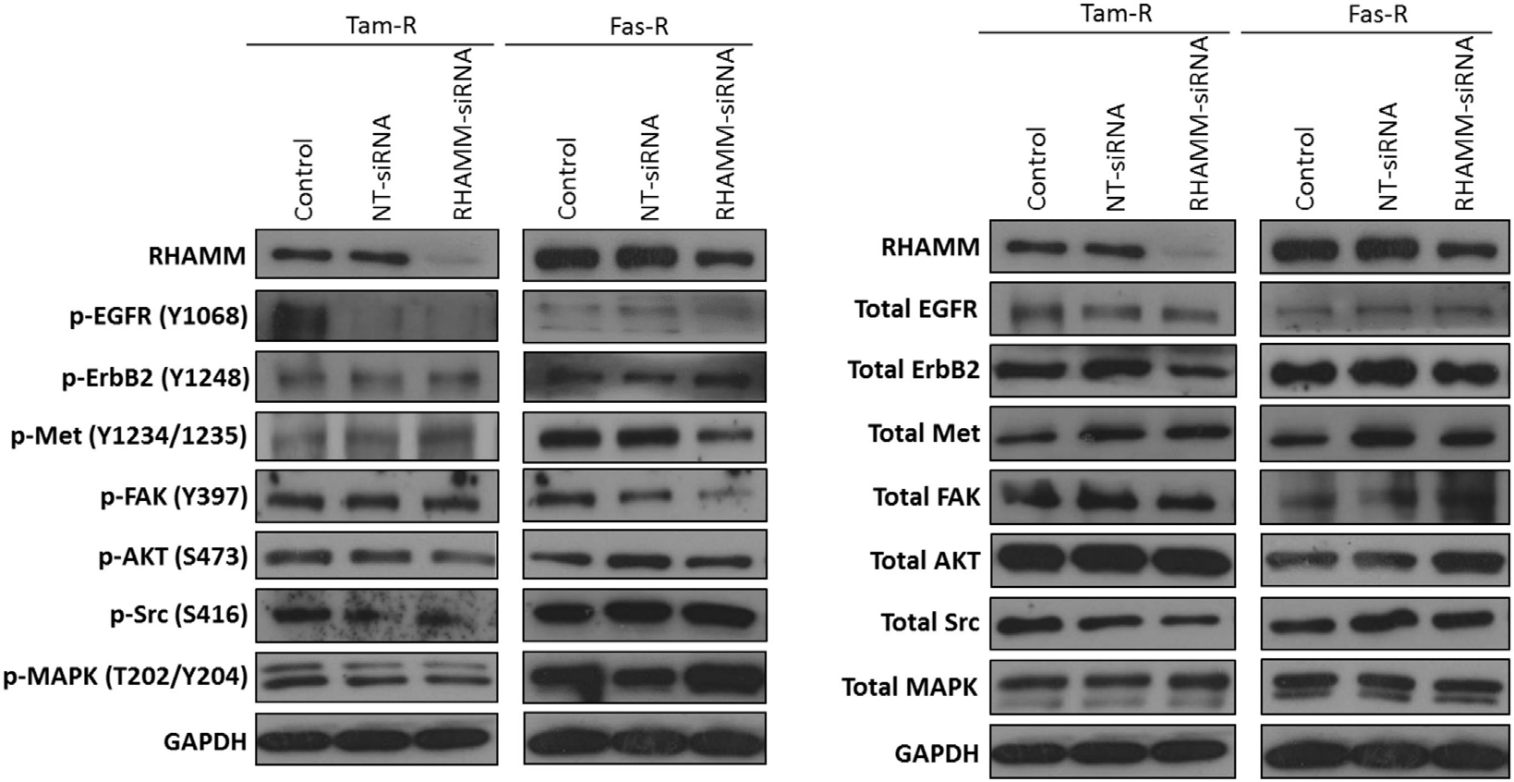
**Effects of RHAMM suppression on pathway signaling**. Western blotting of cells following treatment with RHAMM siRNA revealed that RHAMM suppression did not significantly alter receptor tyrosine kinase and associated pathway signaling in Tam-R cells; however, Fas-R cells showed modest reductions in Met and FAK activity. No change in total protein levels was observed in the absence of RHAMM.

In light of reports that suggest a role for HMMR (RHAMM) in tumor progression (28, 29), we investigated whether RHAMM was contributory toward the endocrine-resistant phenotype of Tam-R and Fas-R cells. Our previous affymetrix data suggested upregulation of RHAMM gene expression in both endocrine-resistant cell models; however, confirmation of RHAMM protein expression by Western blotting revealed a similar level of expression across our cell models (Figure 4A). Immunocytochemistry confirmed these findings and showed slightly reduced RHAMM expression in both endocrine-resistant cells compared to MCF-7 cells and revealed predominant cytoplasmic expression of RHAMM across our cell models (Figure 4B). RHAMM suppression by siRNA did not modulate the expression of CD44 proteins (Figure 4C) and did not alter the aggressive nature of Fas-R cells; interestingly, a reduced level of proliferation was observed in Tam-R cells after RHAMM knockdown (Figures 4D–F). Western blotting of cells following suppression of RHAMM by siRNA revealed that removal of RHAMM did not affect intrinsic receptor tyrosine kinase signaling in Tam-R cells (Figure 5); a similar case was seen for Fas-R cells, although a small reduction was observed in Met and FAK activity.

### Overexpression of CD44v6, but not CD44v3, in MCF-7 Cells Promotes Cellular Invasion and Attenuates Endocrine Response

**Figure 6:**
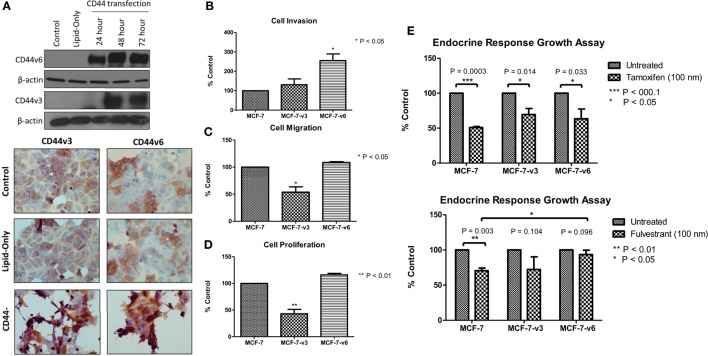
**Overexpression of CD44v6, but not CD44v3, in MCF-7 cells promotes an invasive phenotype**. **(A)** Transfection of MCF-7 cells with CD44v3 or CD44v6 resulted in expression of these proteins to a similar extent as seen in the Tam-R and Fas-R cells but did not alter the expression of other CD44 variants. **(B)** Overexpression of CD44v6 resulted in a gain of invasive capacity in MCF-7 cells, in contrast to CD44v3 overexpression that reduced the migratory **(C)** and proliferative **(D)** capacity of these cells. MCF-7 cells overexpressing CD44 isoforms were exposed to tamoxifen or fulvestrant for 7 days and their growth determined by cell counting. **(E)** CD44v6-overexpressing MCF-7 cells exhibited a reduced response to fulvestrant, but not tamoxifen, compared to the control or CD44v3-overexpressing cells.

Our siRNA data suggest that CD44 *per se* plays an important role in mediating the aggressive phenotype of acquired endocrine-resistant breast cancer cells. However, many CD44 isoforms exist and it is not clear regarding which of these are the dominant contributors in the context of endocrine resistance. To further validate a role for CD44 in the development of an aggressive breast cancer cell phenotype and to begin to explore any differential contribution of CD44 isoforms, we overexpressed CD44v3 and CD44v6, two specific isoforms we have shown to be upregulated in Tam-R and Fas-R cells, separately in MCF-7 cells and assessed any changes to cellular phenotype. Transfection of MCF-7 cells with CD44v3 or CD44v6 resulted in upregulated expression of these isoforms without affecting the expression of other CD44 variants (Figure 6A). Overexpression of CD44v6 resulted in a significant increase in cellular invasion compared to untreated MCF-7 cells; however, this effect was not observed in MCF-7 cells overexpressing CD44v3 (Figure 6B). Interestingly, CD44v3 appeared to also reduce the proliferative and migratory capacity of these cells (Figures 6C,D). Our previous findings suggested that CD44 expression may limit endocrine response in breast cancer cells (23). To investigate this further, specifically in the context of CD44 isoforms, the growth of CD44 isoform-transfected cells was determined in the presence of tamoxifen and fulvestrant. Our data revealed that while CD44v3 overexpression in MCF-7 cells did not significantly alter their response to these agents, overexpression of CD44v6 attenuated the ability of MCF-7 cells to respond to fulvestrant resulting in enhanced proliferative capacity of these cells (Figure 6E). No changes in CD44 variant expression were observed in response to endocrine treatment (data not shown).

### Overexpression of CD44v6 Promotes EGFR Signaling in MCF-7 Cells

**Figure 7:**
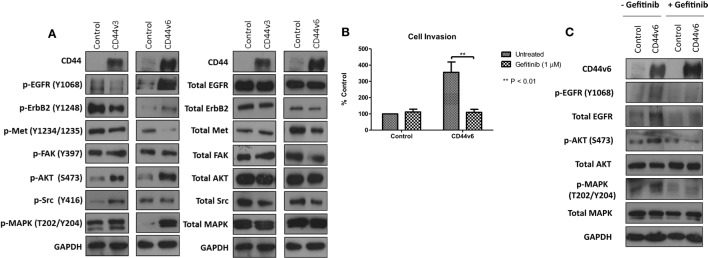
**CD44v6 overexpression enhances EGFR pathway activity in MCF-7 cells**. Western blotting analysis was used to assess the effects of CD44v3 and CD44v6 overexpression in MCF-7 cells on endogenous EGFR and Met pathway activity. **(A)** CD44v6 overexpression in MCF-7 cells significantly reduced endogenous EGFR, AKT, and MAPK phosphorylation, whereas these effects were not seen in MCF-7 cells overexpressing CD44v3; no changes in total protein levels were observed. **(B)** Treatment of CD44v6-overexpressing cells with gefitinib suppressed their invasive behavior together with EGFR pathway activity **(C)**.

Given that our data demonstrated suppression of CD44 inhibited the endogenous activity of the EGFR and Met pathways in our endocrine-resistant cells, we explored the hypothesis that overexpression of CD44 variants in MCF-7 cells might augment growth factor pathway activity. Overexpression of CD44v3 resulted in a modest reduction in ErbB receptor phosphorylation and an increase in AKT and Src activity (Figure 7A). By contrast, MCF-7 cells overexpressing CD44v6 possessed substantially enhanced EGFR, AKT, and MAPK phosphorylation; however, Met activity was reduced. No changes were seen in the level of total proteins expressed. These data suggested that CD44v6 may promote an aggressive, endocrine-desensitized phenotype in MCF-7 cells through the activation of the EGFR pathway. To explore this hypothesis, we investigated the sensitivity of CD44v6-overexpressing MCF-7 cells (MCF-7-CD44v6) to the EGFR inhibitor, gefitinib, with respect to their invasive capacity. Gefitinib inhibited the invasive nature of MCF-7-CD44v6 cells (Figure 7B) and reduced the endogenous activity of EGFR pathway components without affecting total levels of these proteins (Figure 7C).

### Specific Inhibition of CD44v6 in Endocrine-Resistant Cell Models is Associated with a Reduction in Their Invasive Capacity

**Figure 8:**
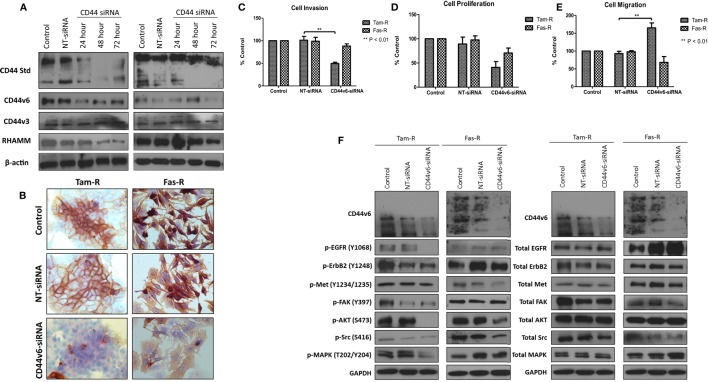
**CD44v6 suppression reduces the invasive behavior of endocrine-resistant breast cancer cells**. **(A,B)** siRNA suppression reduced CD44v6 expression in Tam-R and Fas-R cells without altering the expression of other CD44 variant isoforms. CD44v6 suppression in Tam-R cells reduced their invasive **(C)** and proliferative **(D)** capacity and enhanced their migration **(E)** whereas modest suppression of proliferation and migration was seen in Fas-R cells. **(F)** Western blotting analysis revealed that CD44v6 suppression reduced EGFR pathway component activity in Tam-R cells and suppressed Met and AKT signaling in Fas-R cells but did not alter total protein levels **(F)**.

Having observed a differential contribution to the aggressive phenotype of MCF-7 cells between CD44v3 and CD44v6 overexpression, we extended our studies into the resistant models in which we specifically inhibited CD44v6 expression, using siRNA, to explore the hypothesis that CD44v6 plays a key role in the aggressive phenotype of endocrine resistance. Western blotting revealed that CD44v6 siRNA was specific for this isoform in Tam-R and Fas-R cells (Figures 8A,B). CD44v6 siRNA reduced the endogenous invasive and proliferative capacity of Tam-R but not Fas-R cells (Figures 8C,D). An increase in cellular migration was seen in Tam-R cells following loss of CD44v6 (Figures 8E). Accompanying this was a reduction in EGFR pathway components in Tam-R cells and suppressed Met and AKT signaling in Fas-R cells, however, total protein levels were unaffected (Figure 8F).

### CD44v6 and EGFR Co-Expression Is Associated with a Worsened Outcome in Breast Cancer Patients

**Figure 9:**
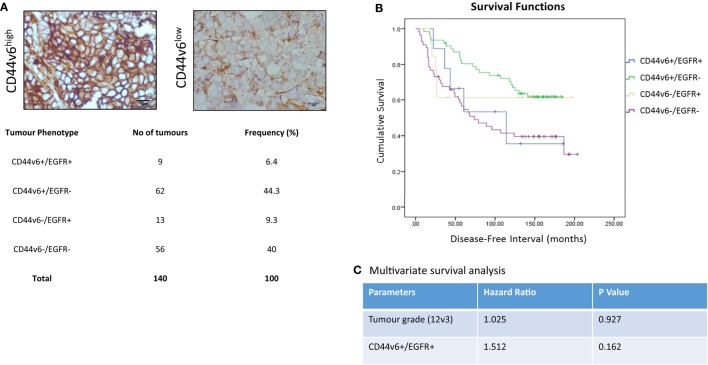
**CD44v6 and EGFR co-expression reduces DFI in tamoxifen-treated ER+ breast cancers**. CD44v6 expression was determined in a TMA series (*n* = 140) of ER+ breast cancer patients that had received tamoxifen and for whom their EGFR status was known. **(A)** Representative images of high (*H*-score >47) and low (*H*-score <47) CD44v6 staining in TMAs and the associated frequency of the four possible phenotypes encountered in this TMA series. **(B)** Kaplan–Meier survival plots for the four possible phenotypes demonstrating that co-expression of CD44v6 and EGFR (or lack of both) were associated with the poorest DFI. **(C)** Cox regression analysis suggested the CD44v6+/EGFR+ phenotype to be more associated with poorer outcome versus grade although this was not significant.

In light of our *in vitro* evidence that CD44v6 represented a potential contributory factor to the aggressive phenotype of endocrine-resistant cells through its ability to modulate EGFR signaling, we explored this hypothesis further in a small exploratory series of formalin-fixed paraffin-embedded TMAs representing 140 patients with ER+ primary breast cancers receiving adjuvant tamoxifen with a 20-year follow-up and for which the EGFR status was already known (30). X-tile was used to define optimal CD44v6 membrane cut point (*H*-score of 47) versus patient outcome and statistical testing was performed using Log Rank (Mantel–Cox). Of the 140 TMA’s studied in this series, 20 were negative for CD44v6 membrane expression. After the cutpoint was applied, 71/140 showed TMAs where CD44v6 staining was greater than the *H*-score cutpoint (Figure 9A). Interestingly, tumors highly positive for CD44v6 but having reduced or absent EGFR appeared to be associated with the most favorable DFI; the impact of co-expression of the EGFR in the CD44v6 cohort was to significantly reduce patient DFI (Figure 9B); Mean survival (months ± SD) = 141.9 ± 7.3 [CD44v6+/EGFR−] versus 105.6 ± 23.4 (CD44v6+/EGFR+), *p* < 0.05. A similar observation was apparent for tumors expressing EGFR in the context of reduced/absent CD44v6 where co-expression resulted in a significantly less favorable DFI than EGFR alone; Mean survival (months ± SD) = 130.0 ± 23.4 [EGFR+/CD44v6−] versus 105.6 ± 23.4 [EGFR+/CD44v6+], *p* < 0.05. Tumors with co-expression of CD44v6 and EGFR had a similar DFI to those absent for both markers. Cox regression (C) revealed that tumors with a CD44v6+/EGFR+ phenotype were more associated with a poorer outcome versus grade although this was not significant.

## Discussion

Although current endocrine treatments for ER+ breast cancers work well, the phenomenon of acquired resistance still represents a major limiting factor in their overall effectiveness and resistant disease frequently occurs at distant sites with associated poor prognosis. Since acquisition of endocrine resistance is accompanied by a gain in aggressive cellular features that may drive distant spread, an understanding of the molecular mechanisms that contribute to this behavior may reveal novel therapeutic targets through which breast cancer outcome can be improved. We have previously shown that the standard form of the cell surface adhesion receptor, CD44, is overexpressed in Tam-R MCF-7 cells. Here, we have extended these investigations to a further model of acquired fulvestrant resistance and also demonstrate the importance of the CD44v6 isoform as a mediator of their invasive phenotype and growth factor (EGFR) signaling in these models.

Our initial investigations revealed that, along with CD44, RHAMM was upregulated in acquired endocrine resistance. Interestingly, while suppression of CD44 by siRNA led to a corresponding reduction in RHAMM in both Tam-R and Fas-R cells, the converse was not seen, i.e., that modulation of RHAMM by siRNA did not affect CD44 expression. Moreover, RHAMM suppression did not affect the invasive, migratory, or proliferative nature of these cells. Taken together, this suggests that the aggressive phenotype in our resistant cell models is not dependent upon RHAMM. Subsequently, we investigated the CD44v3 and v6 isoforms of CD44 given their association with the cancer cell phenotype.

CD44v6 is suggested to represent an important prognostic marker for some cancer types where its elevated expression is associated with metastasis (14, 18). The association between CD44v6 and disease progression may stem from its ability to augment the activity of growth factor signaling pathways. In our study, we investigated changes in the expression and activity of a number of receptor tyrosine kinases known to be linked to an invasive phenotype. Of these, we observed that CD44v6 expression was associated with increased EGFR activity in addition to AKT and MAPK signaling, whereas Met activity was suppressed. Interestingly, Yu et al. (17) found a strong correlation between CD44v6 and AKT expression that was associated with a shorter DFS across 98 samples of breast cancer tissues. Furthermore, AKT activity is reported to be elevated in CD44v6-overexpressing tumors (31), while CD44v6 is able to activate MAPK signaling in combination with other growth factor receptors (32, 33). Our data suggest that CD44v6 expression in some way activates EGFR signaling; we tested this hypothesis by challenging CD44v6+ cells with the EGFR inhibitor, gefitinib. Subsequently, our data showed that gefitinib was able to suppress the invasive capacity of these cells to that of the control levels (Figure 7B) and also prevented the increase in EGFR, AKT, and MAPK observed following CD44v6 overexpression (7C). Given the literature supporting CD44 interactions with other RTKs, there is the possibility that CD44v6 may interact with other receptors in our cell models promoting downstream signaling and an invasive response. However, our data do suggest that irrespective of this, the EGFR represents a dominant pathway given the ability of EGFR pathway inhibition to circumvent CD44v6 effects. Similar findings have been reported by others where interactions between CD44 and erbB members increase cell motility (34–36) while our data additionally point to the co-expression of EGFR and CD44v6 as a clinically relevant phenomenon albeit in a small cohort of patients (Figure 9). Although our Cox regression analysis failed to demonstrate further significance of the CD44v6/EGFR phenotype, this is likely due to the low numbers (*n* = 9) in this subset and further investigation of CD44v6 ± EGFR in a larger number may well reveal a more clearer association with outcome.

Importantly, our data allude to the potential role for CD44v6 in chemoresistance where its overexpression limits cellular response of ER+ endocrine-sensitive cells to fulvestrant. Recent reports have suggested a role between CD44v6 and chemoresistance in prostate cancer which may involve AKT pathway activation (31). Furthermore, a function of CD44v6 as a cell cycle regulator has been suggested potentially through its ability to modulate the Hippo effector, YAP, known to indirectly regulate cell cycle progression (37). Studies that have used combination treatments to suppress cell growth through cell cycle inhibition show that CD44v6 is also reduced in response to these treatments (38). These observations may explain to some degree our observations that CD44v6-overexpressing MCF-7 cells fail to respond to fulvestrant, with CD44v6 providing an indirect, positive input into cell cycle in the presence of fulvestrant. Given the interplay between CD44 and RTKs and the well-established cross talk between RTKs and the ER, it may further be that CD44 upregulation can indirectly activate or modulate the expression of the ER thereby limiting the sensitivity to endocrine agents. While an interesting hypothesis, this did not appear to be the case here as no change in ER expression or activity was seen in MCF-7 or Tam-R cells following CD44 manipulation; Fas-R cells are ER- (39) and no ER restoration was observed following CD44-siRNA (Bellerby, unpublished observations).

While our data suggest an important role for CD44v6 in the aggressive behavior of our endocrine-resistant cells, it is important to note a global knockdown of CD44 expression in both resistant cell models results in differential effects: while Tam-R cells lose their proliferative, invasive, and migratory capacity, only the invasive behavior is affected in Fas-R cells. One explanation for this may be the differential expression of growth factor receptors that accompany different forms of acquired resistance and, thus, provide different interacting partners for the CD44 isoforms. However, that CD44 can be suppressed to attenuate aggressive behavior irrespective of the resistant background suggests that its targeting may be of value.

Whilst CD44 overexpression is widely reported in a number of different tumor types where it correlates with advanced stage/higher grade, such studies invariably use experimental approaches that are unable to differentiate between individual CD44 isoforms, masking the true pattern of CD44 isoform expression. Indeed the fact that some studies suggest a relationship between expression of CD44 standard or its variant isoforms with respect to prognosis (40–42), while other reports are less clear as to a relationship between CD44 and outcome (43–46) is likely due to the complexities of the CD44 expression pattern and the ability to clearly differentiate between different isoforms; our data further point to the importance of understanding the complexities of CD44 variant expression rather than CD44 *per se* in breast cancer. Recent studies have demonstrated the importance of this, showing that a combination of CD44v6 and CD44s, but not CD44 alone, is able to predict survival in lung cancer patients (47), while pancreatic tumors with a “CD44v6+/CD44−” profile are more metastatic (41). Not only will understanding the contribution of specific CD44 isoforms to tumor progression/suppression be beneficial in developing prognostic markers but it may also reveal specific variants that may have therapeutic potential.

The v6 variant of the CD44 family is known to be highly expressed in invasive breast cancers (48) and has itself been the subject of investigation with respect to its therapeutic potential. While in recent years, these approaches have been met with some success in multiple carcinomas, including pancreatic (49), colon (50), and prostate (51) their effectiveness in breast cancer remains limited. However, the identification of CD44 as a marker of breast cancer stem cells has led to the development of a novel strategy through which to target CD44 and, thus, the subpopulation of stem-like cells that may reside in such tumors (52). Recently, the ability to exploit CD44 as a drug-delivery system in cancers, including breast has been investigated with encouraging results (53–55). Thus, the ability to identify specific CD44 isoforms that contribute to the progression of breast cancers offers a potential prognostic and therapeutic target in such tumors. Our data support the role of CD44v6 in breast cancer, particularly in the context of relapsed disease and suggest that therapeutically targeting this isoform may be of benefit in such contexts.

## Author Contributions

RB conceived and performed experimental analysis, data interpretation, manuscript preparation, and review. CS performed experimental analysis and data interpretation. SK undertook immunohistochemical staining of tissue and cell sections. JG undertook critical analysis of microarray data and clinical material data. UG undertook data evaluation and critical review of manuscript. AG undertook statistical analysis and critical review of manuscript. ER undertook review of clinical data and critical review of manuscript. PB-L undertook critical review of manuscript, scientific input, and project direction. SH conceived the project, supervised experimental work, assisted in data interpretation, manuscript preparation, review, and editing.

## Conflict of Interest Statement

This research was conducted in the absence of any commercial or financial relationships that could be construed as a potential conflict of interest.
